# Composition and Long-Term Variation Characteristics of Coral Reef Fish Species in Yongle Atoll, Xisha Islands, China

**DOI:** 10.3390/biology12081062

**Published:** 2023-07-28

**Authors:** Jinfa Zhao, Chunhou Li, Teng Wang, Juan Shi, Xiaoyu Song, Yong Liu

**Affiliations:** 1 Key Laboratory of South China Sea Fishery Resources Exploitation and Utilization, Ministry of Agriculture and Rural Affairs, South China Sea Fisheries Research Institute, Chinese Academy of Fishery Sciences, Guangzhou 510300, China; zhaojf2019@163.com (J.Z.); scslch@vip.163.com (C.L.); sjuan0917@163.com (J.S.); sxy1289667672@163.com (X.S.); 2 Scientific Observation and Research Station of Xisha Island Reef Fishery Ecosystem of Hainan Province, Key Laboratory of Efficient Utilization and Processing of Marine Fishery Resources of Hainan Province, Sanya Tropical Fisheries Research Institute, Sanya 572018, China; 3 Guangdong Provincial Key Laboratory of Fishery Ecology Environment, Guangzhou 510300, China; 4 Observation and Research Station of Pearl River Estuary Ecosystem, Guangzhou 510300, China

**Keywords:** overfishing, habitat decline, individual size, food habits, conservation status

## Abstract

**Simple Summary:**

The coral reef ecosystem not only brings enormous economic value to humans but also provides livelihoods and a major source of protein for millions of people. Moreover, coral reefs provide refuge and food sources for many fish species and are also breeding grounds and spawning grounds for various fish species. However, due to climate and human factors, the coral reef ecosystem has been destroyed, and its ecological function has been damaged. Fishery resources have also been affected, with changes in fish species composition and community structure. This study analyzed the fish community structure of the largest atoll in the Xisha Islands in the South China Sea, summarized a list of coral reef fish in Yongle Atoll, and analyzed the reasons for the change in the fish community and the future variation trend. The completion of this study will contribute to the better protection and recovery of coral reef fish and provide an important reference for the enhancement and restoration of coral reef habitats in the Xisha Islands.

**Abstract:**

Yongle Atoll was the largest atoll in the Xisha Islands of the South China Sea, and it was a coral reef ecosystem with important ecological and economic values. In order to better protect and manage the coral reef fish resources in Yongle Atoll, we analyzed field survey data from artisanal fishery, catches, and underwater video from 2020 to 2022 and combined historical research to explore the changes in fish species composition and community structure in Yongle Atoll over the past 50 years. The results showed that a total of 336 species of fish were found on Yongle Atoll, belonging to 17 orders and 60 families. Among them, Perciformes had the most fish species with 259 species accounting for 77.08% of the total number of species. The number of fish species in the coral reef of Yongle Atoll was exponentially correlated with its corresponding maximum length and significantly decreases with its increase. The fish community structure of Yongle Atoll changed, and the proportion of large carnivorous fish decreased significantly, while the proportion of small-sized and medium-sized fish increased. At the same time, Yongle Atoll has 18 species of fish listed on the IUCN Red List, 15 of which are large fish. The average taxonomic distinctness (Delta+, Δ+) and the variation taxonomic distinctness (Lambda+, Λ+) in 2020–2022 were lower than the historical data, and the number of fish orders, families, and genera in Yongle Atoll has decreased significantly, which indicates that the current coral reef fish species in Yongle Atoll have closer relatives and higher fish species uniformity. In addition, the similarity of fish species in Yongle Atoll was relatively low at various time periods, further proving that the fish community structure has undergone significant variation. In general, due to multiple impacts, such as overfishing, fishing methods, environmental changes, and habitat degradation, the fish species composition of Yongle Atoll may have basically evolved from carnivorous to herbivorous, from large fish to small fish, and from complexity to simplicity, leaving Yongle Atoll in an unstable state. Therefore, we need to strengthen the continuous monitoring of the coral reef ecosystem in Yongle Atoll to achieve the protection and restoration of its ecological environment and fishery resources, as well as sustainable utilization and management.

## 1. Introduction

Coral reefs are one of the most diverse marine ecosystems, known as the “undersea tropical rainforest”, which nourishes one-third of the world’s marine fish and generates enormous economic value for humanity [1]. Coral reef fish provide jobs for 6 million fishermen and direct food sources for hundreds of millions of people [2,3]. However, in recent decades, the health and stability of coral reef ecosystems have been under great pressure, and climate change has gradually become the main threat to coral reef ecosystems. Researchers have studied the changes and causes of coral bleaching events that occur repeatedly on the Great Barrier Reef in Australia and found that coral reefs have little resistance to extremely high temperatures, with global warming being the main factor contributing to their bleaching [4]. Moreover, ocean warming also indirectly threatens coral reefs by increasing the intensity of global cyclones, which can lead to coral reef destruction and associated loss of biological abundance and diversity [5]. Marine pollution caused by human activities poses a great threat to coral reef ecosystems. Plastic waste can be decomposed into microplastics in the ocean, which can enter the body of coral polyps and cause coral reef diseases [6]. In addition, unsustainable human fishing patterns have also caused great damage to the structure of coral reef fish populations. Studies have evaluated the distribution of coral reef fish communities and their interactions with the environment off the coast of East Africa and found that overfishing led to changes in the structure of coral reef fish populations with large fish populations declining rapidly and gradually being replaced by small fish with low economic value [7]. The decline of fish species will also lead to the proliferation of algae and corallivores (such as sea urchins and starfish). The balance between corals and algae will be broken, and a large number of algae will occupy the living space of corals, reducing coral coverage and further degrading coral reef habitats [8].

Yongle Atoll was located in the core area of the western islands of China’s Xisha Islands. It was a typical coral atoll and the largest atoll in the Xisha Islands. The length of the atoll was 24 km from northeast to southwest, the width was 17 km from north to south, and the water depth was about 40 m. The lagoon area was 187.22 km^2^, and the total area was 274 km^2^ (excluding the Money Island reef). The atoll consists of 13 islands, of which, 9 are inhabited islands, including Drummond Island, Yagong Island, Robert Island, Observation Bank, etc. [9,10]. Its abundant fishery resources and high diversity of fish provide refuge and a food source for many fish in the surrounding sea, and it was also the spawning ground and nursery ground for a variety of fish. Since the 1970s, researchers have conducted research on Yongle Atoll, including fish species composition [11], early fish resources [12], fish biology [9], and coral reef community ecology [10]. However, the long-term variation of fish community structure under anthropogenic pressures and climate change was still poorly documented.

Therefore, in this study, we conducted a retrospective analysis of the survey fish data of Yongle Atoll from 1970 to 2022, compiled a list of coral reef fish in Yongle Atoll, analyzed the changes in fish species composition, community structure, feeding habits, and individual size, and analyzed the reasons for this change. This study will provide data for the study of coral reef fisheries in Yongle Atoll, theoretical reference for the protection and management of fisheries and the restoration and protection of coral reefs in the Xisha Islands, and basic data for the study of the geographical distribution of coral reef fishes.

## 2. Materials and Methods

### 2.1. Data Acquisition

**Figure 1:**
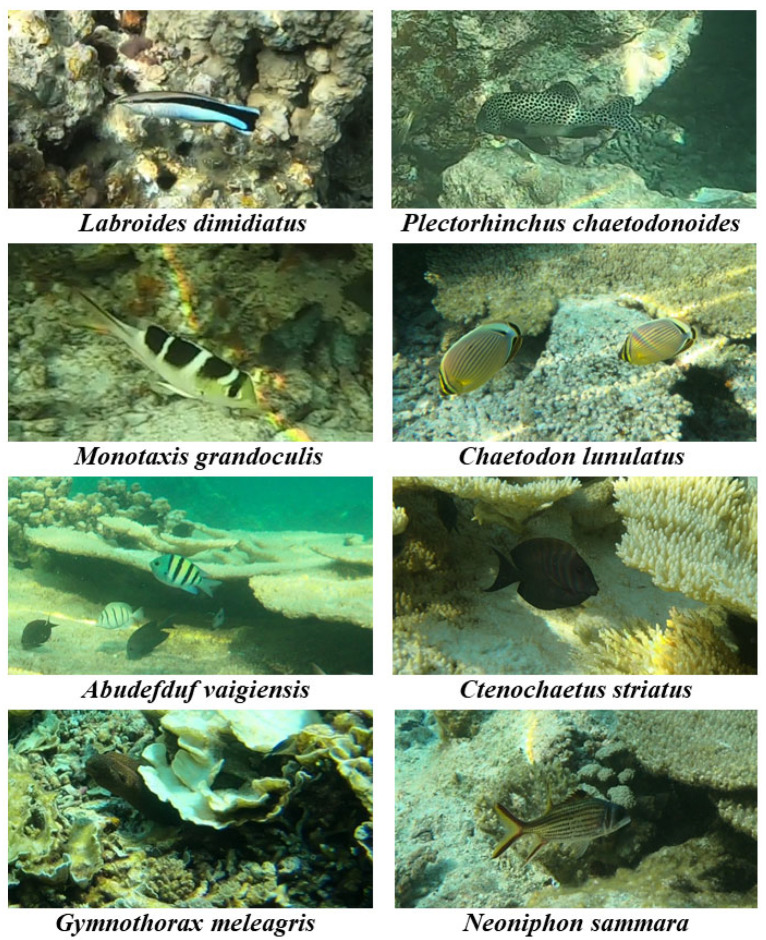
Photos of coral reef fishes extracted from underwater video records of Yongle Atoll.

The data in this study were collected from the historical research of coral reef fishes in Yongle Atoll (16°25′ N–16°36′ N, 111°34′ E–111°48′ E). The historical data of “Ichthyology of South China Sea Islands” [11] in 1970s and the historical archive data of 1998–1999, 2003, and 2005 of the South China Sea Fisheries Research Institute of Chinese Academy of Fishery Sciences were compiled, and the coral reef fish list of Yongle Atoll was formed by combining the field investigation of this study from 2020 to 2022. The survey method used in 1998–1999 was a combination of hand fishing and gill nets. Gill nets were used in the 2003 and 2005 surveys, and nets were generally lowered in the evening and collected in the morning. The survey methods from 2020 to 2021 were diving fishing and underwater video. The diving fishing time was generally from 20:00 to 24:00 in the evening, and the underwater video was conducted during the day. The obtained samples were frozen and kept back in the laboratory for anatomical experiments to preliminarily understand their food classification. The underwater video data were analyzed, identified, and sorted out according to the video image information (Figure 1).

### 2.2. Feeding Habits

Fish feeding habits were divided into three groups, which were carnivorous, herbivorous, and omnivorous. Fish feeding habits comprehensively reference the Fishbase Database (https://www.fishbase.se/search.php, (accessed on 11 May 2023)) [13], the Ecology of Fishes on Coral Reefs [14], and the information on the Ichthyology of the South China Sea Islands [11]. It was found that the clastic feeding recorded by the Fishbase Database had a high overlap with the herbivorous studied by the Ecology of Fishes on Coral Reefs, and it was found that the detritivorous feeding classified by the Fishbase Database was basically herbivorous in anatomy. Therefore, the clastic feeding in the Fishbase Database was classified as herbivorous, and the distinction was determined by referring to the description in the Ichthyology of the South China Sea Islands [11].

### 2.3. Individual Size

The individual size of the fish could be divided into three types, which were large-sized fish: the maximum length was ≥65 cm; medium-sized fish: 65 cm > maximum length ≥ 35 cm; and small-sized fish: maximum length < 35 cm [15]. The maximum total length of fish was obtained from the Fishbase Database, which is the maximum total length of an adult individual of a fish species. And for very few fish that have not obtained the maximum full length, refer to the values for fish of the same genus.

### 2.4. Conservation Status

The conservation status of fish species was based on the International Union for the Conservation of Nature and Natural Resources Red List (IUCN Red List: https://www.iucnredlist.org/, accessed on 11 May 2023) [16]. The protection level of fish species was classified from low to high as Not Evaluated (NE), Data Deficient (DD), Least Concerned (LC), Near Threatened (Near Threatened). NT), Vulnerable (VU), Endangered (EN), Critically Endangered (CR), Extinct in the Wild (EW), and Extinction (EX).

### 2.5. Data Analyses

SPSS 24.0 software was used to analyze the correlation between the maximum length of coral reef fish and the number of species, and linear function, exponential function, power function, and logistic function equation were used to fit, with the largest correlation coefficient R^2^ as the fitting equation to analyze the correlation characteristics. At the same time, an independent sample T-test was used to analyze the significance of differences in the maximum length of coral reef fish of different feeding habits, and the statistical significance criterion was set at 0.05. In addition, Excel 2019 and Origin 2021 were used for data statistical analysis, while Origin 2021 and Photoshop 2019 were used for image processing. The average taxonomic distinctness (Delta+, Δ+) and the variation taxonomic distinctness (Lambda+, Λ+) were used to analyze the diversity of coral reef fish communities [17]. Lambda (+) refers to the theoretical average of the average classification distance path between any pair of species in the species list, which does not change with the number of species. Delta (+) represents the average deviation degree of Lambda (+), represents the degree of difference in path length between species, and reflects the degree of uniform distribution of species composition and kinship. Compared to traditional diversity calculation methods, Lambda (+) and Delta (+) based on taxonomic status can explain the interspecific relationships of communities without being affected by sampling methods and natural changes of habitat ecological types [18]. The taxonomic diversity index Lambda (+) and Delta (+) were calculated using the Taxdtest software package of Primer5.2.

The average taxonomic distinctness (Delta+, Δ+) and the variation taxonomic distinctness (Lambda+, Λ+) [18]:

(1) $$\Delta^{+}=\frac{\sum \sum_{i<j}\omega_{ij}}{S\left(S-1\right)/2}$$

(2) $$\Lambda^{+}=\frac{\sum \sum_{ij}{\left(\omega_{i<j}-\Delta^{+}\right)}^{2}}{S\left(S-1\right)/2}$$

where ω*_ij_* is the path length of the *i* and *j* species in the classification system tree; *S* is the number of species. The weights of the weighted path length among the 6 classification levels of phylum, class, order, family, genus, and species were set as 100.000, 83.333, 66.667, 50.000, 33.333, 16.667.

Jaccard (*J*_s_) [19]:

(3) $$J_{s}=\frac{c}{a+b-c}$$

where *a* is the number of fish species or the number of orders, families, and genera in year *a*; *b* is the number of fish species or the number of orders, families, and genera in year *b*; *c* is the number of fish species or the number of orders, families, and genera in common in the two years investigated. The similarity level was as follows: 0 < *J*_s_ < 0.25 was extremely dissimilar; 0.25 ≤ *J*_s_ < 0.50 was not similar; 0.50 ≤ *J*_s_ < 0.75 was moderate similarity; 0.75 ≤ *J*_s_ < 1.00 was extremely similar.

Coral Fish Diversity Index (CFDI) [20]:

*N* = 3.39(CFDI) − 20.595 (4)

where *N* is the predicted total number of species, and CFDI is the total number of species in the six families of Acanthuridae, Chaetodontidae, Labridae, Scaridae, Pomacanthidae, and Pomacentridae.

## 3. Results

### 3.1. Fish Species Composition

**Figure 2:**
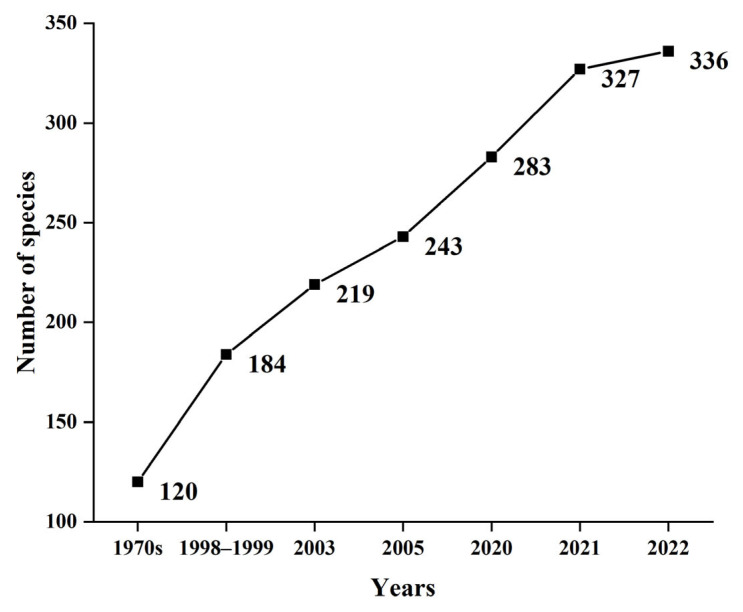
Temporal variation of fish species on the coral reef fishes of Yongle Atoll.

Based on historical data and archival data of the South China Sea Fisheries Research Institute, this study shows that a total of 336 fish species have been found in Yongle Atoll (Figure 2, Table S1). The composition of fish species found in the surveys has increased significantly from the 1970s to 2022, with 123 more species in 2005 than in the 1970s and 93 more in 2022 than in 2005. Overall, the increase in fish species composition in each survey showed a downward trend. In particular, only nine new species were discovered in 2022 compared with 2021, which is a sharp decline from the previous data.

**Figure 3:**
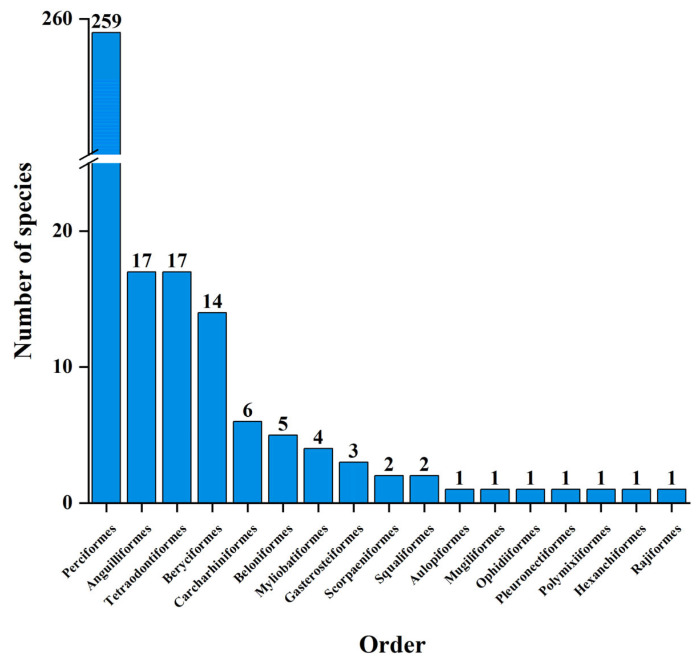
The distribution characteristics of species number at the order level of coral reef fishes in Yongle Atoll.

**Figure 4:**
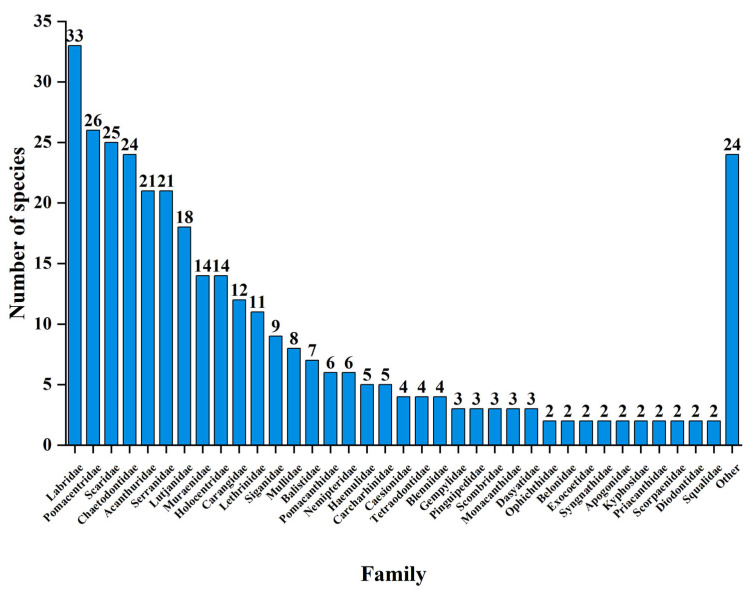
The distribution characteristics of species number at the family level of coral reef fishes in Yongle Atoll.

The coral reef fish in Yongle Atoll belong to the order Actinopterygii (322 species) and the order Chondrichthyes (14 species), consisting of 17 orders, namely, Perciformes, Anguilliformes, Tetraodontiformes, Beryciformes, Carcharhiniformes, Beloniformes, Myliobatiformes, Gasterosteiformes, Scorpaeniformes Squaliformes, Aulopiformes, Mugiliformes, Ophidiiformes, Pleuronectiformes, Polymixiiformes, Hexanchiformes, and Rajiformes. Among them, the Perciformes has the highest number of fish species with 259 species accounting for 77.08% of the total, followed by 17 species in the order Anguilliformes, 17 species in the order Tetraodontiformes, 14 species in the order Beryciformes, and less than 10 species in other orders (Figure 3). The coral reef fish in Yongle Atoll were composed of 60 families, among which, Labridae has the highest number of species, accounting for 33 species. The following were Pomacentridae (26 species), Scaridae (25 species), Chaetodontidae (24 species), Acanthuridae (21 species), Serranidae (21 species), Lutjanidae (18 species), Muraenidae (14 species), Holocentridae (14 species), Carangidae (12 species), and Lethrinidae (11 species), while the number of species in other families was lower than 10 (Figure 4).

**Figure 5:**
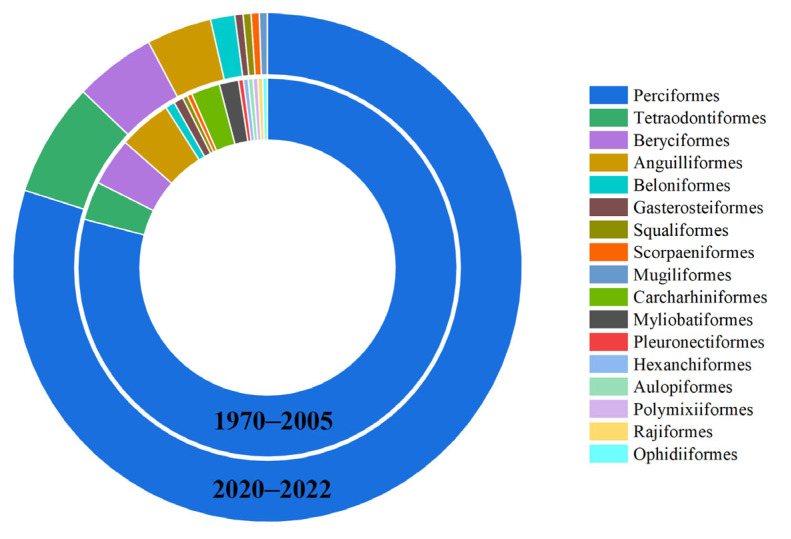
The distribution characteristics of species number at the order level of coral reef fishes in Yongle Atoll in different years.

**Figure 6:**
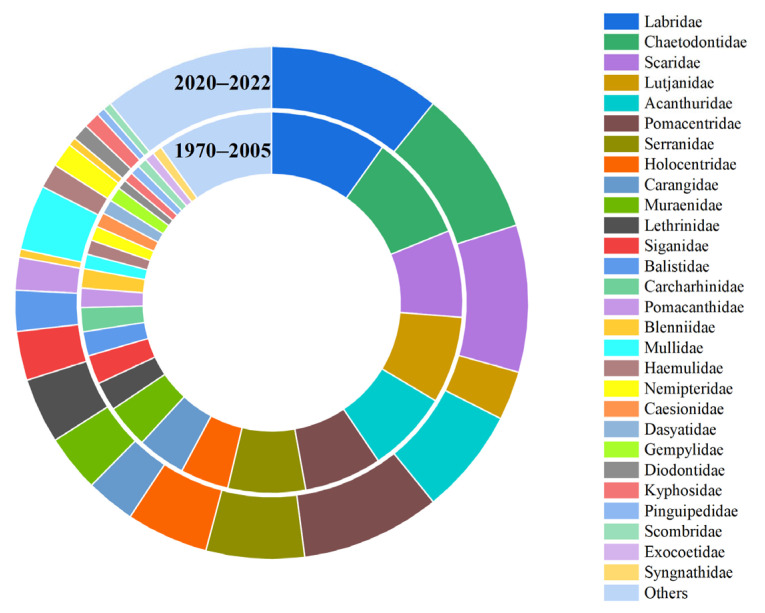
The distribution characteristics of species number at the family level of coral reef fishes in Yongle Atoll in different years.

In the past 50 years, although Perciformes were the first dominant order in both periods, the composition of orders also showed significant changes, among which, the proportion of Perciformes increased to 79.10% (193 species) during 1970–2005 and 79.90% (155 species) during 2020–2022 (Figure 5). During 2020–2022, compared to 1970–2005, Carcharhiniformes, Myliobatiformes, Pleuronectiformes, Hexanchiformes, Aulopiformes, Polymixiiformes, Rajiformes, and Ophidiiformes did not reappear, while Mugiliformes emerged. During the two periods, the second dominant order of fishes also showed changes, from Anguilliformes (4.51%) during 1970–2005 to Tetraodontiformes (7.22%) during 2020–2022 and from carnivorous fishes to omnivorous fishes (Figure 5). In both periods, the dominant family of fish in Yongle Atoll was Labridae, and its proportion increased from 9.84% (24 species) during 1970–2005 to 10.82% (21 species) during 2020–2022. Compared to 1970–2005, there were 22 families that did not reappear in 2020–2022, while 9 new families emerged. Among the top 20 most species-rich families in both periods, Carcharhinidae, Blenniidae, and Caesionidae lost their positions in the top 20 during 1970–2005 and were replaced by Diodontidae, Tetraodontidae, and Monacanthidae (Figure 6). In the past 50 years, the changes in fish composition in Yongle Atoll have basically presented the variation trend of miniaturization and “vegetarianization”.

### 3.2. Fish Community Structure and Changes

**Figure 7:**
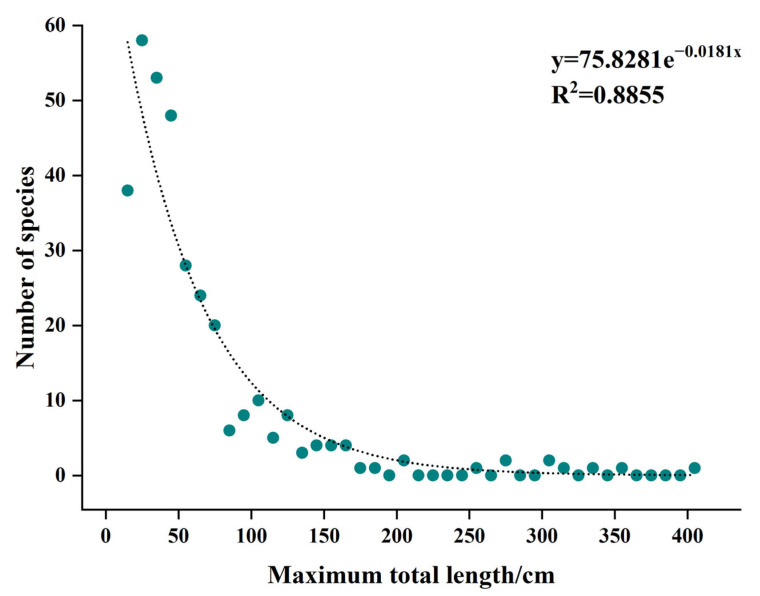
The distribution characteristics of species number at the maximum total length of coral reef fishes in Yongle Atoll.

The maximum total length of coral reef fish in Yongle Atoll was distributed from 3.3 cm to 750 cm. The maximum total length of coral reef fish in Yongle Atoll was divided from 0 cm into an interval of 10 cm to calculate the number of species in each interval, and the median represented the maximum total length of this interval. There were no more than 1 species in the interval above 400 cm, and only 2 groups of intervals had species distribution. Therefore, these outliers were discarded in the analysis of species numbers with maximum total length distribution. The overall results showed that there was an exponential correlation between the number of species and their maximum total length. When the maximum total length was less than 120 cm, the number of species decreased significantly with the increase of the maximum total length, while when the maximum total length was greater than 120 cm, the number of species decreased slowly with the increase of the maximum total length. The results showed that the fish species in Yongle Atoll were mainly concentrated in the interval with a maximum total length of 0–120 cm, and the number of fish species in this interval was up to 299, accounting for 88.99% of the fish species in Yongle Atoll (Figure 7).

**Figure 8:**
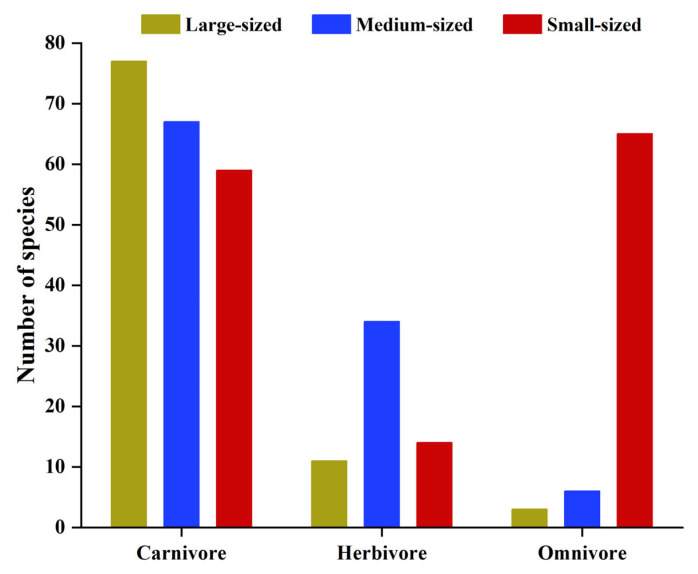
Distribution characteristics of coral reef fishes with different sizes and feeding habits in Yongle Atoll.

In terms of body size, Yongle Atoll has 138 species of small-sized fish, accounting for 41.07% of the total number of fish species. Next came 107 species of medium-sized fishes, accounting for 31.85% of the total. There were 91 species of large-sized fish, accounting for 27.08% of the total. There was little difference in the number of fish species of different body types, and the whole fish group was mainly small-sized fish and medium-sized fish. In terms of feeding types, the number of carnivorous fish species was 203, accounting for 60.42% of the total fish species. Next were 74 species of omnivorous fish, accounting for 22.02% of the total, and 59 species of herbivorous fish, accounting for 17.56% of the total. Carnivorous fishes dominated the large-sized and medium-sized fish groups, accounting for 84.62% and 62.62%, respectively, followed by herbivorous fishes, accounting for 12.09% and 31.78%, respectively, and omnivorous fishes were the least, accounting for 3.30% and 5.61%, respectively. Among the small-sized fishes, omnivorous and carnivorous fish occupy an absolute advantage, accounting for 47.10% and 42.75% of their respective groups, respectively, and herbivorous fish were the least, accounting for only 10.14% (Figure 8).

**Figure 9:**
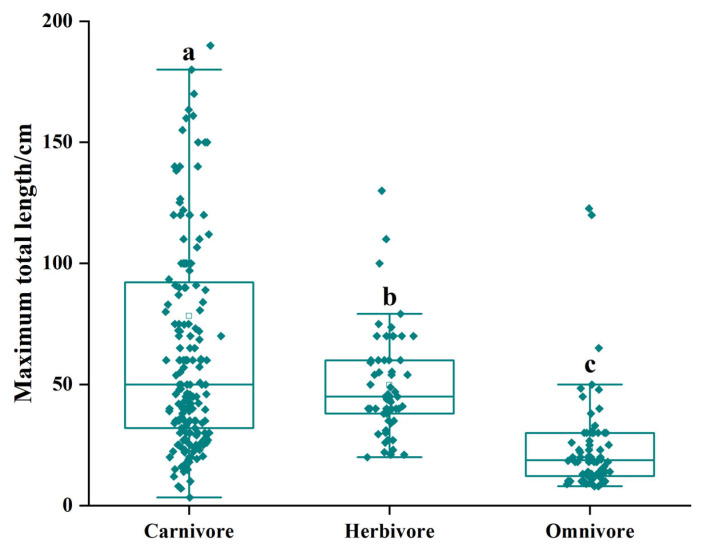
Body size characteristics of coral reef fishes with different feeding habits in Yongle Atoll. (a, b, c: different letters indicate significant differences.)

In carnivorous fish, the distribution of the three different sizes is more uniform, while medium-sized fish dominate in the herbivorous fish. Omnivorous fish were dominated by small-sized fish; the proportion was as high as 87.84% (Figure 8). The maximum total length distribution range of the three feeding habits of fish was significantly different. The average total length of the carnivorous fish was 76.8 ± 86.10 cm, and the distribution range was the largest, ranging from 3.32–750.00 cm. The individual omnivorous fish was the smallest with an average maximum total length of 23.6 ± 19.81 cm and a distribution range of 8.00–122.70 cm. The average maximum total length of phytophagous fish was 48.4 ± 21.47 cm, and the distribution range was minimal, ranging from 20.00–130.00 cm (Figure 9).

**Figure 10:**
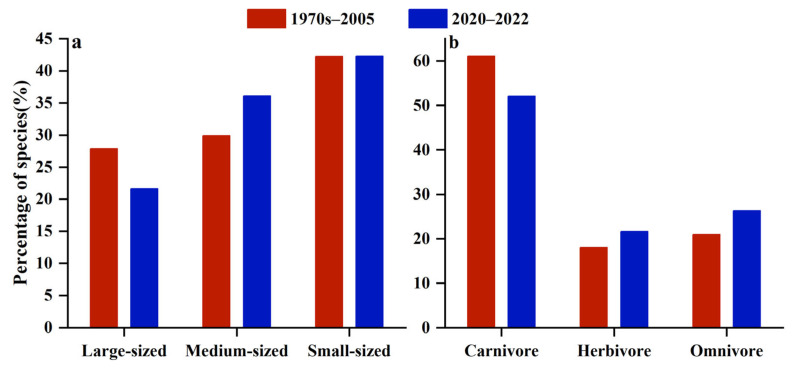
Historical comparative distribution characteristics of coral reef fishes of different sizes and feeding habits in Yongle Atoll. ((**a**). Different individual sizes, (**b**). Different feeding habits.)

In the current survey (2020–2022), the highest proportion of small-sized fish in Yongle Atoll was 42.29% with a small increase compared to the historical data (1970s–2005). The proportion of large-sized fish was 21.65%, which changed obviously compared to the history, and the number of species decreased by 56, and the proportion decreased by 6.22%. The proportion of medium-sized fish was 36.08%, an increase of 6.16% compared to the historical level (Figure 10A). At the same time, the number of carnivorous fish species decreased significantly, decreasing by 58 species (9.00%) compared to the historical number, while the proportion of herbivorous fish and omnivorous fish increased by 3.62% and 5.39% compared to the historical data (Figure 10B). Moreover, compared to the historical data, the number of orders, families, and genera of coral reef fish in Yongle Atoll has significantly decreased, with the number of objects reduced by 7, the number of families reduced by 13, and the number of genera decreased by 43. In addition, the Lambda+(Λ+) and Delta+(Δ+) of coral reef fish in Yongle Atoll during 1970–2005 were 165.7 and 56.83, respectively. The Lambda+(Λ+) and Delta+(Δ+) values for coral reef fish in 2020–2022 were 114.7 and 54.85.

From the perspective of similarity, the similarity index between the different time periods was relatively low. The similarity of order, family, genus, and species between the 1970s and 1998–2005 was 0.17, 0.22, 0.12, and 0.10, respectively. The similarity of order, family, genus, and species between 1970s and 2020–2022 was 0.25, 0.29, 0.28, and 0.22, respectively. The similarity of order, family, genus, and species between 1998–2005 and 2020–2022 were 0.27, 0.31, 0.24, and 0.21, respectively (Table 1).

### 3.3. Disappearing Fish Community Structure

Compared to the historical survey, 142 species of fish were not found in this study, especially carnivorous fish (102 species), accounting for 71.83% of the total number of undiscovered fish followed by herbivorous and omnivorous fish 11.97% (17 species) and 16.20% (23 species), respectively. Moreover, all three different sizes of fish are predominantly carnivorous; 91.55% of these undiscovered fish belong to the order Perciformes, Anguilliformes, Beryciformes, Tetraodontiformes, Carcharhiniformes, and Myliobatiformes.

**Figure 11:**
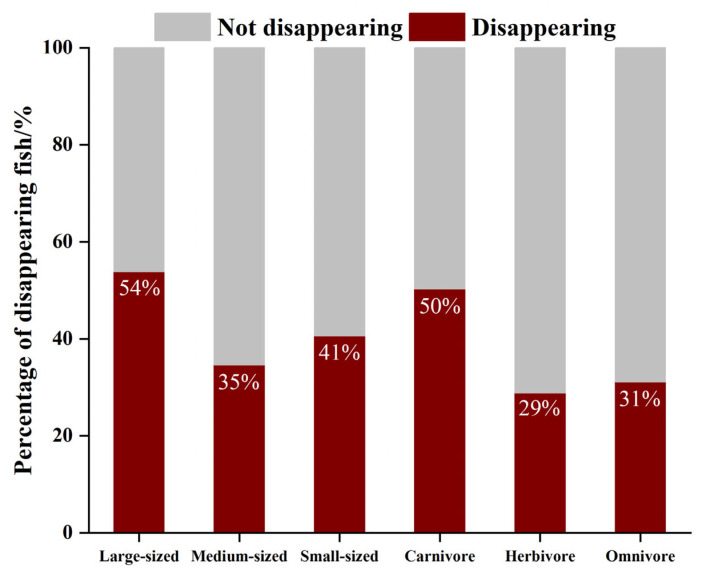
The proportion of the disappearing coral reef fishes in each functional group in Yongle Atoll.

**Figure 12:**
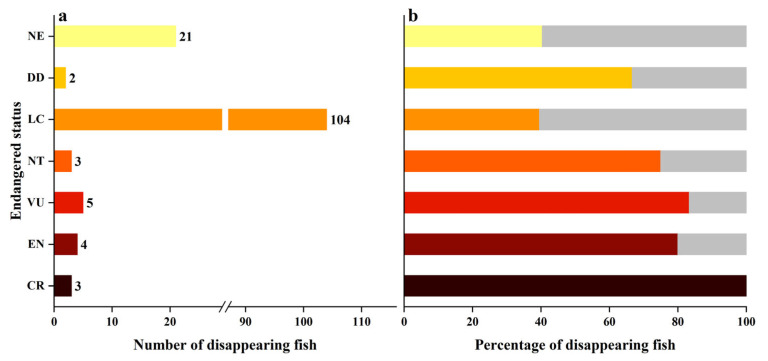
The Conservation state of the disappearing coral reef fishes in Yongle Atoll. ((**a**). Number of disappearing fish, (**b**). Percentage of disappearing fish.)

There was a total of 142 fish species that have not been rediscovered on the coral reef of Yongle Atoll, accounting for 42.26% of the total fish species. In terms of different body types, the disappearance rate of large-sized fish was the highest (53.85%) followed by small-sized fish (40.58%), and the disappearance rate of medium-sized fish was the lowest (34.58%). In terms of different feeding habits, the disappearance rate of carnivorous fish was the highest, accounting for 50.25% of the total number of carnivorous fish, followed by omnivorous fish, accounting for 31.08% of the total number of omnivorous fish, and the disappearance rate of herbivorous fish was the lowest, accounting for 28.81% of the total number of herbivorous fish (Figure 11). In addition, 18 species of fish were listed on the IUCN Red List in the coral reef of Yongle Atoll, of which, 15 species have not been rediscovered. There were three critically endangered species, *Carcharhinus longimanus*, *Rhynchobatus djiddensis*, and *Glyphis gangeticus*, which have not been found again. There were five endangered species, namely, *Cheilinus undulatus*, *Squalus japonicus*, *Mustellus griseus*, *Dasyatis sinensis*, and *Squalus brevirostris*. Among them, four species have not been rediscovered. Vulnerable species include six, including *Carcharhinus falciformis*, *Taeniura meyeni*, *Urugymnus asperrmius*, *Epinephelus fuscoguttatus*, and *Bolbometoton muriatum*, among which, five have not been found again. There were four species near threatened, namely, *Galeocerdo cuvier*, *Hexanchus griseus*, *Carcharhinus amblyrhynchoides*, and *Chaetodon trifascialis*. Three of them have not been found again (Figure 12). The above fish species, except for *Bolbometoton muriatum*, *Chaetodon trifascialis*, and *Squalus japonicus*, have not been found, and *Squalus japonicus* was a newly discovered fish in Yongle Atoll.

## 4. Discussion

### 4.1. Causes of Changes in Fish Species Composition

This study combined historical data to summarize the coral reef fish species in Yongle Atoll and found that there were a relatively rich number of 336 species of coral reef fish in Yongle Atoll. Compared with other islands of the Xisha Islands in the South China Sea, it was relatively rich in fish species (such as Dongdao Island (235 species) [21], Qilianyu Islands (315 species) [15], Yongxing Island (341 species) [22]), probably because Yongle Atoll has open lagoons, complex coral reef structures, more feeding microhabitats, greater off-reef transport rates, and more reliable food access. Moreover, it was more suitable as a spawning ground and nursery ground for fish [12,23]. At the same time, according to Allen’s Coral Fish Diversity Index (CFDI) [20], which predicts the total species of coral reef fish in Yongle Atoll, including 6 families, including Acanthuridae, Chaetodontidae, Labridae, Scaridae, Pomacanthidae, and Pomacentridae, the total species number was 3.39 (CFDI)-20.595, resulting in a possible 437 species of coral reef fish in Yongle Atoll. Nearly 101 species have not been discovered, possibly due to their extinction or extremely low resource levels. In addition, the composition of the fish community in Yongle Atoll has undergone significant changes in the past 50 years, with the Anguilliformes gradually losing their second dominant position and being replaced by the Tetraodontiformes. Anguilliformes were typical cave-dwelling fish that have certain requirements for the three-dimensional structure of coral reefs. Their extinction may be caused by the destruction of the three-dimensional structure of coral reefs, habitat decline, or overfishing. Moreover, the species similarity in several historical periods of this study was particularly low, indicating that the fish succession and changes in Yongle Atoll were very significant, which also preliminarily supports the above results.

The coral reef waters of the Indian Pacific Ocean were extremely rich in coral reef fish, inhabiting over 100 families of fish. The research shows that 29 families of fish cover most of these coral reef fish, which were Serranidae, Chaetodontidae, Labridae, Scaridae, Gobiidae, Acanthuridae, Pomacentridae, Mullidae, Siganidae, Lutjanidae, Haemulidae, Lethrinidae, Apogonidae, Pseudochoromidae, Cirrhitidae, Pomacanthidae, Carangidae, Blenniidae, Holocentridae, Tetraodontidae, Balistidae, Nemipteridae, Monacanthidae, Scorpaenidae, Muraenidae, Syngnathidae, Pinguipedidae, Caesinidae, and Microdesmidae [24]. In this study, it was found in all the other families except Gobiidae and Microdesmidae, which accounted for 85.1 of the total species. The results of this study were similar to other islands of the Xisha Islands in the South China Sea. For example, 4 families were not found on Dongdao Island, and the remaining families accounted for 82.13% of the total species [21]. A total of 1 family was not found in the Qilianyu islands, and the rest accounted for 89.52% of the total species [15]. However, the proportion of these 29 families of coral reef fish to the total species was higher than that of Redang Islands in Malaysia (63.50%) [25], Mayotte Island in the Southwest Indian Ocean (73.80%) [26], and Ouvea Atoll in New Caledonia (79.60%) [27]. The average taxonomic distinctness (Delta+, Δ+) reflects the proximity of relatives between fish communities [28]. Compared to the historical study (1970–2005), the average taxonomic distinctness (Delta+, Δ+) of coral reef fish in Yongle Atoll in 2020–2022 was smaller, indicating that the coral reef fish in 2020–2022 were more closely related. The variation in taxonomic distinctness (Lambda+, Λ+) reflects the evenness of the taxonomic relationship, which was similar to the variation trend of the average taxonomic distinctness. Compared to historical studies (1970–2005), the coral coverage and habitat diversity of Yongle Atoll were significantly reduced, and the lack of environmental heterogeneity and stability reduced the opportunities for the coexistence of different species [29]. Therefore, the variation taxonomic distinctness (Lambda+, Λ+) decreased; that is, the uniformity of species taxonomic relationship among fish communities increased. Similar evolution trends also appeared in Terminos Lagoon [30] in the Gulf of Mexico and the Mauritanian coast [31] in the northwest of the Atlantic Ocean, and the taxonomic diversity index of fish communities showed a monotonous downward trend. In addition, the number of fish orders, families, and genera in Yongle Atoll has decreased significantly, which further verifies the above view. These all indicate that the current fish species have closer relatives and the evenness of fish species was higher, which will lead to the increase of instability of the ecosystem and reduce the resilience of the ecosystem [32,33] and further prove the necessity of monitoring and protection of their habitat changes.

### 4.2. Causes of Changes in Fish Community Structure

After years of long-term investigation, the species composition of coral reef fish in Yongle Atoll can basically represent the original state of this area. The coral reef fishes in Yongle Atoll were overwhelmingly carnivorous, which was consistent with the earlier research that most coral reef fishes were carnivorous [34]. However, the current survey found that compared to the historical data, carnivore fish had the most obvious changes. Among the undiscovered fish, carnivore fish accounted for the highest proportion, reaching 71.83%. The nutrition pyramid changed to a low trophic level but did not reverse directly, which was consistent with the rule that fish communities were initially disturbed [35]. In addition, 15 of the 18 kinds of fish listed in the IUCN Red List were the top carnivore fish in the ocean, which further indicates that carnivore fish were more likely to die out, which was in line with the law that the current fishery activities lead to the decline of the trophic level of the ecosystem. At the same time, both herbivorous and omnivorous fishes have increased to a certain extent compared to previous studies, especially the number of omnivorous fishes, which indicates that the coral reef ecosystem was in the process of succession from carnivorous fishes to herbivorous fishes. The results of this study were similar to those of other islands and reefs in the Xisha Islands (Qilianyu Islands [15], Yongxing Island [22], and Dongdao Island [21]) in the South China Sea, which was consistent with the succession process of coral reef fish in the Xisha Islands. A similar evolution has also occurred in coral reefs in Tanzania [36], Australia’s Great Barrier Reef [37], and the Caribbean [38]. The change in the coral reef ecosystem will lead to an increase in herbivorous fish. In the short term, the change of fishery fishing to herbivorous fish has significant value for fishery discovery, but in the long term, overfishing will destroy the balance and function of the ecosystem [39].

Under the influence of climate and human activities, large-sized coral reef fish will decline and die out first. Because large fish have high economic value and are the main target species for fishing, the characteristics of large fish, such as slow growth and late sexual maturity, make it difficult to quickly replenish when the population is impacted [40,41]. This study just verified this theory; the highest proportion of large-sized fish disappearing among Yongle Atoll reef fish was 53.85%, while small-sized and medium-sized fish have increased. There were three possible explanations for the fact that smaller fish were better adapted to the current reef environment than larger fish. First, small fish have a higher population turnover rate and speciation rate, shorter life cycles, high reproduction rate, and abundant complementary groups, which can better adapt to the fragmentation of coral reef habitats [42]. Second, small fish live in a small range, their long-distance swimming ability is weak, the demand for food resources is low, and the same area can accommodate more small species [43]. Third, environmental factors can also affect the size of fish. Studies have analyzed the reproductive biological characteristics of female *Larimichthys polyactis* in the Yellow Sea and Bohai Sea from 1960 to 2020 and found that in addition to fishing pressure, the rise in water temperature was also the reason for the early sexual maturity and size reduction of *Larimichthys polyactis* [44]. It was worth noting that small-sized fish also account for a large proportion of undiscovered fish, especially reef fish. Most of these fish are highly dependent on the coral reef ecosystem, and some can only survive in the living coral habitat. The complex reef structure not only provides refuge for coral reef fish but also provides rich food for them [45,46]. Therefore, the decline of habitat may directly lead to the loss of habitat for these small reef-dwelling fish and lead to extinction. Coral cover in the Xisha Islands has also declined significantly in recent years [47]. In addition, we also found traces of *Acanthaster planci* in the underwater video, which also confirmed that the Yongle Atoll habitat was declining. To prevent further deterioration of the fish composition in the coral reef of Yongle Atoll, it was necessary to control the local fishing intensity, monitor and understand changes in its habitat, and take necessary measures to prevent further decline in this habitat.

Yongle Atoll was the largest atoll in the Xisha Islands. Affected by a large number of human activities in recent years, the species diversity of fish was generally declining, and the succession of fish composition was obvious. The main reasons for this phenomenon were as follows: Firstly, overfishing was a direct factor leading to a decrease in fish abundance and changes in community structure [48]. With the rapid development of marine fish, fishing techniques have become more progressive, and fishing intensity has increased year by year and far exceeds the replenishment capacity of marine fish resources, causing changes in fish community structure [49]. The research results of some studies and the Ecological Environment Bulletin of Hainan Province show that the density of fish in the coral reef of the Xisha Islands will decline from 310 fish/100 m^2^ in 2005 to 146.7 fish/100 m^2^ in 2021, further supporting the impact of human activities on fish in the islands [47]. Secondly, the change in fishing methods, especially selective fishing, was a key factor leading to changes in fish community structure. Large carnivorous fish were the key fishing objects of the fishery, while a large number of non-economic or low-economic value fish would not be selected [40,50]. As a result, large coral reef fish were more likely to be caught than smaller fish [51]. The apparent decline of large carnivorous fish in the Qilianyu Islands [15], Dongdao Island [21], and Yongxing Island [22] in the Xisha Islands, as well as reefs in Australia’s Great Barrier Reef [37] and the Caribbean [38], further supports this view. Thirdly, there were changes in marine environmental factors, and ocean warming was a factor that affects fish diversity and resource levels. Rising water temperature can alter the rhythm of feeding, digestion, and movement activities of marine fish and reduce their reproductive capacity, as fish need to consume additional energy to cope with extreme temperature events [52,53]. Studies have used the Ecoath with Ecosim model to explore the impact of ocean warming on the coral reef ecosystem of the Xisha Islands in the South China Sea. The results showed that by the middle of this century, ocean warming will lead to a decline of 3.79% in total catch compared to 2009 [54]. Moreover, the sea surface temperature of the Xisha Islands rose faster than that of the Zhongsha and Nansha Islands [55]. Fourthly, the habitat of fish has been destroyed. Coral reefs provide habitats for a large number of marine fish, but with the increasing severity of human activities and climate change, a large number of coral reefs have undergone bleaching and death and have lost their three-dimensional structure, thereby losing their function as fish habitats, leading to the disappearance of a large number of marine fish [56,57,58]. Changes in the fish community in Australia’s Great Barrier Reef over a period of 15 years following habitat degradation and restoration have been studied and found that the richness of fish communities was consistent with the trend of coral species restoration [46]. These results indicate that the restoration of coral communities and the complex interaction of coral reef frameworks determine the functional structure of related fish communities and further demonstrate the importance of coral reef habitats for fish communities.

In order to better understand and grasp the evolution dynamics of fish communities in the Yongle Atoll area in the future, it is necessary to increase the frequency of surveys as much as possible while protecting the ecological environment and use a unified survey method to obtain more high-quality continuous observation data. At the same time, combined with long-term historical data, the evolution trend of the fish community structure was analyzed from a long-term scale analysis to provide a scientific basis for sustainable development of fishery resources and policy management.

## 5. Conclusions

This study analyzed the evolution of the coral reef fish community structure in Yongle Atoll over the past 50 years and compiled a relatively complete list of coral reef fish in Yongle Atoll. At present, the coral reef fish in Yongle Atoll were mainly affected by factors such as overfishing, habitat degradation, and rising water temperature, resulting in a decrease in the richness and diversity of coral reef fish, changes in the community structure, and obvious succession of fish composition. This study was of great significance for the protection and restoration of coral reef fish in Yongle Atoll and provides an important reference for the enhancement and restoration of the coral reef habitat in the Xisha Islands.

## Figures and Tables

**Table 1:** Fish species similarity at Yongle Atoll in different years.

Years	1998–2005				2020–2022			
	Order	Family	Genus	Species	Order	Family	Genus	Species
1970s	0.17	0.22	0.12	0.10	0.25	0.29	0.28	0.22
1998–2005	/	/	/	/	0.27	0.31	0.24	0.21

## Data Availability

Part of the data presented in this study are available in the Supplementary Material. The remaining data presented in this study are available upon reasonable request from the corresponding author.

## Supplementary Materials

The following supporting information can be downloaded at: https://www.mdpi.com/article/10.3390/biology12081062/s1, Table S1: The checklist of coral reef fishes in Yongle Atoll.
