# Supplementary material for: Composition and Long-Term Variation Characteristics of Coral Reef Fish Species in Yongle Atoll, Xisha Islands, China
Source: Biology (Basel). 2023 Jul 28;12(8):1062. doi: 10.3390/biology12081062 (PMC10451685; doi:10.3390/biology12081062)
Supplement: Supplementary file 1 [file biology-12-01062-s001.zip › biology-2472202-supplementary.pdf]

Table S1: The checklist of coral reef fishes in Yongle Atoll.

No.	Class, Order, Family, Genus, Species	IUCN	Feeding habits	Habitat
	**Actinopterygii**			
	**Anguilliformes**			
	Congridae			
	**Conger**			
1	*Conger japonicus*	NE	Carnivore	DEM
	Muraenidae			
	**Echidna**			
2	*Echidna nebulosa*	LC	Carnivore	RFA
3	*Echidna polyzona*	LC	Carnivore	RFA
	**Gymnothorax**			
4	*Gymnothorax albimarginatus*	LC	Carnivore	RFA
5	*Gymnothorax fimbriatus*	LC	Carnivore	BRA;RFA
6	*Gymnothorax isingteena*	LC	Carnivore	RFA
7	*Gymnothorax javanicus*	LC	Carnivore	RFA
8	*Gymnothorax meleagris*	LC	Carnivore	BRA;RFA
9	*Gymnothorax neglectus*	LC	Carnivore	DEM
10	*Gymnothorax pictus*	LC	Carnivore	BRA;RFA
11	*Gymnothorax richardsonii*	LC	Carnivore	RFA
12	*Gymnothorax rueppellii*	LC	Carnivore	BRA;RFA
13	*Gymnothorax thyrsoideus*	LC	Carnivore	RFA
14	*Gymnothorax undulatus*	LC	Carnivore	BRA;RFA
	**Scuticaria**			
15	*Scuticaria tigrina*	LC	Carnivore	RFA
	Ophichthidae			
	**Myrichthys**			
16	*Myrichthys colubrinus*	NE	Carnivore	RFA
17	*Myrichthys maculosus*	NE	Carnivore	RFA
	**Aulopiformes**			
	Synodontidae			
	**Synodus**			
18	*Synodus variegatus*	LC	Carnivore	RFA
	**Beloniformes**			
	Belonidae			
	**Strongylura**			
19	*Strongylura incisa*	NE	Carnivore	RFA
	**Tylosurus**			
20	*Tylosurus melanotus*	NE	Carnivore	BRA;RFA;OD
	Exocoetidae			
	**Cheilopogon**			
21	*Cheilopogon cyanopterus*	LC	Carnivore	PELO;OD
	**Parexocoetus**			

No.	Class, Order, Family, Genus, Species	IUCN	Feeding habits	Habitat
22	*Parexocoetus brachypterus*	NE	Carnivore	PE;OD
	Hemiramphidae			
	**Hyporhamphus**			
23	*Hyporhamphus dussumieri*	NE	Carnivore	RFA
	**Beryciformes**			
	Holocentridae			
	**Myripristis**			
24	*Myripristis kuntee*	LC	Carnivore	RFA
25	*Myripristis murdjan*	LC	Carnivore	RFA
26	*Myripristis pralinia*	LC	Carnivore	RFA
27	*Myripristis violacea*	LC	Carnivore	RFA
28	*Myripristis vittata*	LC	Carnivore	RFA
	**Neoniphon**			
29	*Neoniphon opercularis*	LC	Carnivore	RFA
30	*Neoniphon sammara*	LC	Carnivore	RFA
	**Ostichthys**			
31	*Ostichthys kaianus*	LC	Carnivore	BAD
	**Sargocentron**			
32	*Sargocentron caudimaculatum*	LC	Carnivore	RFA
33	*Sargocentron diadema*	LC	Carnivore	RFA
34	*Sargocentron ensifer*	LC	Carnivore	RFA
35	*Sargocentron melanospilos*	LC	Carnivore	RFA
36	*Sargocentron punctatissimum*	LC	Carnivore	RFA
37	*Sargocentron spiniferum*	LC	Carnivore	RFA
	**Gasterosteiformes**			
	Fistulariidae			
	**Fistularia**			
38	*Fistularia commersonii*	LC	Carnivore	RFA
	Syngnathidae			
	**Corythoichthys**			
39	*Corythoichthys flavofasciatus*	LC	Carnivore	RFA
	**Doryrhamphus**			
40	*Doryrhamphus excisus*	LC	Carnivore	RFA
	**Mugiliformes**			
	Mugilidae			
	**Oedalechilus**			
41	*Oedalechilus labiosus*	NE	Omnivore	RFA
	**Ophidiiformes**			
	Carapidae			
	**Encheliophis**			
42	*Encheliophis homei*	LC	Carnivore	RFA
	**Perciformes**			
	Acanthuridae			

No.	Class, Order, Family, Genus, Species	IUCN	Feeding habits	Habitat
	**Acanthurus**			
43	*Acanthurus dussumieri*	LC	Herbivore	RFA
44	*Acanthurus gahhm*	LC	Herbivore	RFA
45	*Acanthurus japonicus*	LC	Herbivore	RFA
46	*Acanthurus lineatus*	LC	Herbivore	RFA
47	*Acanthurus mata*	LC	Herbivore	RFA
48	*Acanthurus nigrofuscus*	LC	Herbivore	RFA
49	*Acanthurus olivaceus*	LC	Herbivore	RFA
50	*Acanthurus thompsoni*	LC	Herbivore	BRA;RFA
51	*Acanthurus triostegus*	LC	Herbivore	RFA
52	*Acanthurus xanthopterus*	LC	Herbivore	RFA
	**Ctenochaetus**			
53	*Ctenochaetus binotatus*	LC	Herbivore	RFA
54	*Ctenochaetus striatus*	LC	Herbivore	RFA
	**Naso**			
55	*Naso annulatus*	LC	Herbivore	RFA
56	*Naso brevirostris*	LC	Herbivore	RFA
57	*Naso hexacanthus*	LC	Herbivore	BRA;RFA
58	*Naso lituratus*	LC	Herbivore	RFA
59	*Naso thynnoides*	LC	Herbivore	RFA
60	*Naso unicornis*	LC	Herbivore	RFA
61	*Naso vlamingii*	LC	Herbivore	RFA
	**Zebrasoma**			
62	*Zebrasoma flavescens*	LC	Herbivore	RFA
63	*Zebrasoma velifer*	LC	Herbivore	RFA
	Apogonidae			
	**Cheilodipterus**			
64	*Cheilodipterus quinquelineatus*	NE	Carnivore	RFA
	**Gymnapogon**			
65	*Gymnapogon philippinus*	NE	Carnivore	RFA
	Blenniidae			
	**Aspidontus**			
66	*Aspidontus tractus*	LC	Omnivore	RFA
	**Blenniella**			
67	*Blenniella periophthalmus*	LC	Omnivore	RFA
	**Istiblennius**			
68	*Istiblennius edentulus*	LC	Omnivore	BRA;RFA
	**Salarias**			
69	*Salarias fasciatus*	LC	Omnivore	BRA;RFA
	Caesionidae			
	**Caesio**			
70	*Caesio caerulaurea*	LC	Carnivore	RFA
71	*Caesio lunaris*	LC	Carnivore	RFA

No.	Class, Order, Family, Genus, Species	IUCN	Feeding habits	Habitat
	**Pterocaesio**			
72	*Pterocaesio digramma*	LC	Carnivore	RFA
73	*Pterocaesio tile*	LC	Carnivore	RFA
	Carangidae			
	**Carangoides**			
74	*Carangoides ferdau*	LC	Carnivore	BRA;RFA
	**Caranx**			
75	*Caranx ignobilis*	LC	Carnivore	BRA;RFA
76	*Caranx lugubris*	LC	Carnivore	BEP;OD
77	*Caranx melampygus*	LC	Carnivore	BRA;RFA
78	*Caranx sexfasciatus*	LC	Carnivore	BRA;RFA;AMP
	**Decapterus**			
79	*Decapterus macrosoma*	LC	Carnivore	RFA
	**Scomberoides**			
80	*Scomberoides lysan*	LC	Carnivore	BRA;RFA
	**Selar**			
81	*Selar crumenophthalmus*	LC	Carnivore	RFA
	**Selaroides**			
82	*Selaroides leptolepis*	LC	Carnivore	BRA;RFA;AMP
	**Seriola**			
83	*Seriola lalandi*	LC	Carnivore	BRA;BEP
84	*Seriola quinqueradiata*	LC	Carnivore	DEM;OD
	**Trachinotus**			
85	*Trachinotus baillonii*	LC	Carnivore	BRA;RFA
	Chaetodontidae			
	**Chaetodon**			
86	*Chaetodon auriga*	LC	Omnivore	RFA
87	*Chaetodon auripes*	LC	Omnivore	RFA
88	*Chaetodon citrinellus*	LC	Omnivore	RFA
89	*Chaetodon ephippium*	LC	Omnivore	RFA
90	*Chaetodon falcula*	LC	Omnivore	RFA
91	*Chaetodon kleinii*	LC	Omnivore	RFA
92	*Chaetodon lineolatus*	LC	Omnivore	RFA
93	*Chaetodon lunula*	LC	Omnivore	RFA
94	*Chaetodon lunulatus*	LC	Omnivore	RFA
95	*Chaetodon madagaskariensis*	LC	Omnivore	RFA
96	*Chaetodon melannotus*	LC	Omnivore	RFA
97	*Chaetodon ornatissimus*	LC	Omnivore	RFA
98	*Chaetodon rafflesii*	LC	Omnivore	BRA;RFA
99	*Chaetodon semeion*	LC	Omnivore	RFA
100	*Chaetodon speculum*	LC	Omnivore	RFA
101	*Chaetodon trifascialis*	NT	Omnivore	RFA
102	*Chaetodon trifasciatus*	LC	Omnivore	RFA

No.	Class, Order, Family, Genus, Species	IUCN	Feeding habits	Habitat
103	*Chaetodon unimaculatus*	LC	Omnivore	RFA
104	*Chaetodon vagabundus*	LC	Omnivore	RFA
105	*Chaetodon wiebeli*	LC	Omnivore	RFA
	**Forcipiger**			
106	*Forcipiger flavissimus*	LC	Omnivore	RFA
107	*Forcipiger longirostris*	LC	Carnivore	RFA
	**Heniochus**			
108	*Heniochus acuminatus*	LC	Omnivore	BRA;RFA
109	*Heniochus chrysostomus*	LC	Omnivore	RFA
110	*Heniochus singularius*	LC	Omnivore	RFA
	Cirrhitidae			
	**Cirrhitus**			
111	*Cirrhitus pinnulatus*	LC	Carnivore	RFA
	Ephippidae			
	**Platax**			
112	*Platax orbicularis*	LC	Carnivore	BRA;RFA
	Gempylidae			
	**Rexea**			
113	*Rexea prometheoides*	NE	Carnivore	BEP
	**Ruvettus**			
114	*Ruvettus pretiosus*	LC	Carnivore	BEP;OD
	**Thyrsitoides**			
115	*Thyrsitoides marleyi*	NE	Carnivore	BEP
	Gerreidae			
	**Gerres**			
116	*Gerres filamentosus*	LC	Carnivore	BRA;DEM;AMP
	Haemulidae			
	**Plectorhinchus**			
117	*Plectorhinchus chaetodonoides*	NE	Carnivore	BRA;RFA
118	*Plectorhinchus diagrammus*	NE	Carnivore	RFA
119	*Plectorhinchus lineatus*	NE	Carnivore	RFA
120	*Plectorhinchus picus*	NE	Carnivore	RFA
121	*Plectorhinchus vittatus*	LC	Carnivore	RFA
	Kyphosidae			
	**Kyphosus**			
122	*Kyphosus cinerascens*	LC	Herbivore	RFA
123	*Kyphosus vaigiensis*	LC	Herbivore	RFA;OD
	Labridae			
	**Anampses**			
124	*Anampses caeruleopunctatus*	LC	Carnivore	RFA
	**Bodianus**			
125	*Bodianus axillaris*	LC	Carnivore	RFA
126	*Bodianus macrourus*	LC	Carnivore	RFA

No.	Class, Order, Family, Genus, Species	IUCN	Feeding habits	Habitat
127	*Bodianus oxycephalus*	DD	Carnivore	RFA
	**Cheilinus**			
128	*Cheilinus chlorourus*	LC	Carnivore	RFA
129	*Cheilinus fasciatus*	LC	Carnivore	RFA
130	*Cheilinus trilobatus*	LC	Carnivore	BRA;RFA
131	*Cheilinus undulatus*	EN	Carnivore	RFA
	**Coris**			
132	*Coris gaimard*	LC	Carnivore	RFA
	**Cymolutes**			
133	*Cymolutes lecluse*	LC	Carnivore	RFA
	**Epibulus**			
134	*Epibulus insidiator*	LC	Carnivore	RFA
	**Gomphosus**			
135	*Gomphosus varius*	LC	Carnivore	RFA
	**Halichoeres**			
136	*Halichoeres chloropterus*	LC	Carnivore	RFA
137	*Halichoeres hortulanus*	LC	Carnivore	RFA
138	*Halichoeres marginatus*	LC	Carnivore	RFA
139	*Halichoeres prosopeion*	LC	Carnivore	RFA
140	*Halichoeres trimaculatus*	LC	Carnivore	RFA
	**Hemigymnus**			
141	*Hemigymnus fasciatus*	LC	Carnivore	RFA
142	*Hemigymnus melapterus*	LC	Carnivore	RFA
	**Hologymnosus**			
143	*Hologymnosus annulatus*	LC	Carnivore	RFA
	**Labroides**			
144	*Labroides dimidiatus*	LC	Carnivore	RFA
	**Macropharyngodon**			
145	*Macropharyngodon meleagris*	LC	Carnivore	RFA
	**Novaculichthys**			
146	*Novaculichthys taeniourus*	LC	Carnivore	RFA
	**Oxycheilinus**			
147	*Oxycheilinus celebicus*	LC	Carnivore	RFA
148	*Oxycheilinus digramma*	LC	Carnivore	BRA;RFA
149	*Oxycheilinus unifasciatus*	LC	Carnivore	RFA
	**Stethojulis**			
150	*Stethojulis bandanensis*	LC	Carnivore	RFA
151	*Stethojulis strigiventer*	LC	Carnivore	RFA
	**Thalassoma**			
152	*Thalassoma amblycephalum*	LC	Carnivore	RFA
153	*Thalassoma hardwicke*	LC	Carnivore	RFA
154	*Thalassoma lunare*	LC	Carnivore	RFA
155	*Thalassoma purpureum*	LC	Carnivore	RFA

No.	Class, Order, Family, Genus, Species	IUCN	Feeding habits	Habitat
156	*Thalassoma quinquevittatum*	LC	Carnivore	RFA
	Lethrinidae			
	**Gnathodentex**			
157	*Gnathodentex aureolineatus*	LC	Carnivore	RFA
	**Gymnocranius**			
158	*Gymnocranius griseus*	LC	Carnivore	RFA
	**Lethrinus**			
159	*Lethrinus erythropterus*	LC	Carnivore	RFA
160	*Lethrinus harak*	LC	Carnivore	BRA;RFA
161	*Lethrinus miniatus*	LC	Carnivore	BRA;RFA
162	*Lethrinus obsoletus*	LC	Carnivore	RFA
163	*Lethrinus olivaceus*	LC	Carnivore	RFA
164	*Lethrinus rubrioperculatus*	LC	Carnivore	RFA
165	*Lethrinus variegatus*	LC	Carnivore	RFA
166	*Lethrinus xanthochilus*	LC	Carnivore	RFA
	**Monotaxis**			
167	*Monotaxis grandoculis*	LC	Carnivore	RFA
	Lutjanidae			
	**Aphareus**			
168	*Aphareus furca*	LC	Carnivore	RFA
169	*Aphareus rutilans*	LC	Carnivore	RFA
	**Aprion**			
170	*Aprion virescens*	LC	Carnivore	RFA
	**Etelis**			
171	*Etelis carbunculus*	LC	Carnivore	BEP
	**Lutjanus**			
172	*Lutjanus argentimaculatus*	LC	Carnivore	BRA;RFA;OD
173	*Lutjanus bohar*	LC	Carnivore	BRA;RFA
174	*Lutjanus fulviflamma*	LC	Carnivore	BRA;RFA
175	*Lutjanus fulvus*	LC	Carnivore	BRA;RFA
176	*Lutjanus gibbus*	LC	Carnivore	RFA
177	*Lutjanus kasmira*	LC	Carnivore	RFA
178	*Lutjanus monostigma*	LC	Carnivore	BRA;RFA
179	*Lutjanus stellatus*	NE	Carnivore	RFA
	**Macolor**			
180	*Macolor niger*	LC	Carnivore	RFA
	**Paracaesio**			
181	*Paracaesio sordida*	LC	Carnivore	RFA
182	*Paracaesio xanthura*	LC	Carnivore	RFA
	**Pristipomoides**			
183	*Pristipomoides auricilla*	LC	Carnivore	BEP
184	*Pristipomoides filamentosus*	LC	Carnivore	BEP
	**Symphorichthys**			

No.	Class, Order, Family, Genus, Species	IUCN	Feeding habits	Habitat
185	*Symphorichthys spilurus*	LC	Carnivore	RFA
	Malacanthidae			
	**Malacanthus**			
186	*Malacanthus brevirostris*	NE	Carnivore	RFA
	Mullidae			
	**Mulloidichthys**			
187	*Mulloidichthys flavolineatus*	LC	Carnivore	RFA
188	*Mulloidichthys vanicolensis*	LC	Carnivore	RFA
	**Parupeneus**			
189	*Parupeneus barberinus*	LC	Carnivore	RFA
190	*Parupeneus cyclostomus*	LC	Carnivore	RFA
191	*Parupeneus indicus*	LC	Carnivore	BRA;RFA
192	*Parupeneus multifasciatus*	LC	Carnivore	RFA
193	*Parupeneus pleurostigma*	LC	Carnivore	RFA
194	*Parupeneus trifasciatus*	LC	Carnivore	RFA
	Nemipteridae			
	**Pentapodus**			
195	*Pentapodus aureofasciatus*	LC	Carnivore	RFA
196	*Pentapodus caninus*	LC	Carnivore	RFA
197	*Pentapodus nagasakiensis*	LC	Carnivore	BRA;RFA
	**Scolopsis**			
198	*Scolopsis aurata*	LC	Carnivore	RFA
199	*Scolopsis lineata*	LC	Carnivore	RFA
200	*Scolopsis trilineata*	LC	Carnivore	RFA
	Pempheridae			
	**Pempheris**			
201	*Pempheris mangula*	NE	Carnivore	DEM
	Pentacerotidae			
	**Histiopterus**			
202	*Histiopterus typus*	NE	Carnivore	RFA
	Pinguipedidae			
	**Parapercis**			
203	*Parapercis cylindrica*	NE	Carnivore	RFA
204	*Parapercis hexophtalma*	NE	Carnivore	RFA
205	*Parapercis pacifica*	NE	Carnivore	BEP
	Plesiopidae			
	**Plesiops**			
206	*Plesiops coeruleolineatus*	LC	Carnivore	RFA
	Pomacanthidae			
	**Centropyge**			
207	*Centropyge bispinosa*	LC	Omnivore	RFA
208	*Centropyge tibicen*	LC	Omnivore	RFA
209	*Centropyge vrolikii*	LC	Omnivore	RFA

No.	Class, Order, Family, Genus, Species	IUCN	Feeding habits	Habitat
	**Pomacanthus**			
210	*Pomacanthus imperator*	LC	Omnivore	RFA
211	*Pomacanthus semicirculatus*	LC	Omnivore	RFA
	**Pygoplites**			
212	*Pygoplites diacanthus*	LC	Omnivore	RFA
	Pomacentridae			
	**Abudefduf**			
213	*Abudefduf septemfasciatus*	LC	Omnivore	RFA
214	*Abudefduf sordidus*	LC	Herbivore	BRA;RFA
215	*Abudefduf vaigiensis*	LC	Omnivore	RFA;OD
	**Amblyglyphidodon**			
216	*Amblyglyphidodon aureus*	LC	Omnivore	RFA
	**Amphiprion**			
217	*Amphiprion clarkii*	NE	Omnivore	RFA
218	*Amphiprion frenatus*	LC	Omnivore	RFA
219	*Amphiprion perideraion*	LC	Omnivore	BRA;RFA
	**Chromis**			
220	*Chromis margaritifer*	NE	Omnivore	RFA
221	*Chromis retrofasciata*	NE	Omnivore	RFA
222	*Chromis viridis*	NE	Omnivore	RFA
	**Chrysiptera**			
223	*Chrysiptera biocellata*	NE	Omnivore	RFA
224	*Chrysiptera brownriggii*	NE	Omnivore	RFA
	**Dascyllus**			
225	*Dascyllus aruanus*	NE	Omnivore	RFA
226	*Dascyllus melanurus*	NE	Omnivore	RFA
227	*Dascyllus trimaculatus*	NE	Omnivore	RFA
	**Dischistodus**			
228	*Dischistodus perspicillatus*	NE	Omnivore	BRA;RFA
	**Hemiglyphidodon**			
229	*Hemiglyphidodon plagiometopon*	NE	Omnivore	RFA
	**Neoglyphidodon**			
230	*Neoglyphidodon thoracotaeniatus*	NE	Omnivore	RFA
	**Plectroglyphidodon**			
231	*Plectroglyphidodon dickii*	NE	Omnivore	RFA
232	*Plectroglyphidodon lacrymatus*	NE	Omnivore	RFA
	**Pomacentrus**			
233	*Pomacentrus ambionensis*	NE	Omnivore	RFA;OD
234	*Pomacentrus coelestis*	NE	Omnivore	RFA
235	*Pomacentrus pavo*	NE	Omnivore	RFA
236	*Pomacentrus tripunctatus*	NE	Omnivore	BRA;RFA
	**Stegastes**			
237	*Stegastes fasciolatus*	NE	Omnivore	RFA

No.	Class, Order, Family, Genus, Species	IUCN	Feeding habits	Habitat
238	*Stegastes lividus*	NE	Omnivore	RFA
	Priacanthidae			
	**Heteropriacanthus**			
239	*Heteropriacanthus cruentatus*	LC	Carnivore	RFA
	**Priacanthus**			
240	*Priacanthus hamrur*	LC	Carnivore	RFA
	Pseudochromidae			
	**Pseudochromis**			
241	*Pseudochromis fuscus*	LC	Omnivore	RFA
	Scaridae			
	**Bolbometopon**			
242	*Bolbometopon muricatum*	VU	Herbivore	RFA
	**Calotomus**			
243	*Calotomus carolinus*	LC	Herbivore	RFA
244	*Calotomus japonicus*	LC	Herbivore	RFA
245	*Calotomus spinidens*	LC	Herbivore	RFA
	**Cetoscarus**			
246	*Cetoscarus bicolor*	LC	Herbivore	RFA
	**Chlorurus**			
247	*Chlorurus microrhinos*	LC	Herbivore	RFA
248	*Chlorurus sordidus*	LC	Herbivore	BRA;RFA;OD
	**Hipposcarus**			
249	*Hipposcarus longiceps*	LC	Herbivore	RFA
	**Scarus**			
250	*Scarus chameleon*	LC	Herbivore	RFA
251	*Scarus dimidiatus*	LC	Herbivore	BRA;RFA
252	*Scarus ferrugineus*	LC	Herbivore	RFA
253	*Scarus festivus*	LC	Herbivore	BRA;RFA
254	*Scarus forsteni*	LC	Herbivore	RFA
255	*Scarus frenatus*	LC	Herbivore	RFA
256	*Scarus ghobban*	LC	Herbivore	BRA;RFA
257	*Scarus globiceps*	LC	Herbivore	RFA
258	*Scarus niger*	LC	Herbivore	RFA
259	*Scarus oviceps*	LC	Herbivore	RFA
260	*Scarus prasiognathos*	LC	Herbivore	RFA
261	*Scarus psittacus*	LC	Herbivore	RFA
262	*Scarus rivulatus*	LC	Herbivore	RFA
263	*Scarus rubroviolaceus*	LC	Herbivore	RFA
264	*Scarus schlegeli*	LC	Herbivore	RFA
265	*Scarus tricolor*	LC	Herbivore	RFA
	Scombridae			
	**Grammatorcynus**			
266	*Grammatorcynus bicarinatus*	LC	Carnivore	RFA;OD

No.	Class, Order, Family, Genus, Species	IUCN	Feeding habits	Habitat
	**Gymnosarda**			
267	*Gymnosarda unicolor*	LC	Carnivore	RFA;OD
	**Rastrelliger**			
268	*Rastrelliger kanagurta*	DD	Carnivore	PE;OD
	Serranidae			
	**Aethaloperca**			
269	*Aethaloperca rogaa*	LC	Carnivore	RFA
	**Cephalopholis**			
270	*Cephalopholis argus*	LC	Carnivore	RFA
271	*Cephalopholis leopardus*	LC	Carnivore	RFA
272	*Cephalopholis sonnerati*	LC	Carnivore	RFA
273	*Cephalopholis urodeta*	LC	Carnivore	RFA
	**Epinephelus**			
274	*Epinephelus coioides*	LC	Carnivore	BRA;RFA
275	*Epinephelus cyanopodus*	LC	Carnivore	RFA
276	*Epinephelus fasciatus*	LC	Carnivore	BRA;RFA
277	*Epinephelus fuscoguttatus*	VU	Carnivore	RFA
278	*Epinephelus hexagonatus*	LC	Carnivore	RFA
279	*Epinephelus latifasciatus*	LC	Carnivore	DEM
280	*Epinephelus maculatus*	LC	Carnivore	RFA
281	*Epinephelus merra*	LC	Carnivore	RFA
282	*Epinephelus poecilonotus*	LC	Carnivore	BRA;RFA
283	*Epinephelus retouti*	LC	Carnivore	RFA
284	*Epinephelus tauvina*	DD	Carnivore	RFA;OD
285	*Epinephelus trimaculatus*	LC	Carnivore	RFA
	**Plectropomus**			
286	*Plectropomus leopardus*	LC	Carnivore	RFA
287	*Plectropomus oligacanthus*	LC	Carnivore	RFA
	**Variola**			
288	*Variola albimarginata*	LC	Carnivore	RFA
289	*Variola louti*	LC	Carnivore	RFA
	Siganidae			
	**Siganus**			
290	*Siganus argenteus*	LC	Herbivore	RFA
291	*Siganus corallinus*	LC	Herbivore	RFA
292	*Siganus fuscescens*	LC	Herbivore	BRA;RFA;OD
293	*Siganus puellus*	LC	Herbivore	RFA
294	*Siganus punctatissimus*	LC	Herbivore	RFA
295	*Siganus stellatus*	LC	Herbivore	RFA
296	*Siganus vermiculatus*	LC	Herbivore	BRA;RFA
297	*Siganus virgatus*	LC	Herbivore	BRA;RFA
298	*Siganus vulpinus*	LC	Herbivore	RFA
	Sparidae			

No.	Class, Order, Family, Genus, Species	IUCN	Feeding habits	Habitat
	**Acanthopagrus**			
299	*Acanthopagrus berda*	LC	Carnivore	BRA;DEM;OD
	Sphyraenidae			
	**Sphyraena**			
300	*Sphyraena forsteri*	NE	Carnivore	RFA
	Zanclidae			
	**Zanclus**			
301	*Zanclus cornutus*	LC	Herbivore	RFA
	**Pleuronectiformes**			
	Bothidae			
	**Bothus**			
302	*Bothus pantherinus*	LC	Carnivore	RFA
	**Polymixiiformes**			
	Polymixiidae			
	**Polymixia**			
303	*Polymixia berndti*	LC	Carnivore	RFA
	**Scorpaeniformes**			
	Scorpaenidae			
	**Dendrochirus**			
304	*Dendrochirus bellus*	LC	Carnivore	DEM
	**Synanceia**			
305	*Synanceia verrucosa*	LC	Carnivore	RFA
	**Tetraodontiformes**			
	Balistidae			
	**Balistapus**			
306	*Balistapus undulatus*	NE	Omnivore	RFA
	**Balistoides**			
307	*Balistoides viridescens*	NE	Carnivore	RFA
	**Melichthys**			
308	*Melichthys vidua*	NE	Omnivore	RFA
	**Rhinecanthus**			
309	*Rhinecanthus aculeatus*	NE	Omnivore	RFA
310	*Rhinecanthus rectangulus*	NE	Omnivore	RFA
	**Sufflamen**			
311	*Sufflamen chrysopterum*	NE	Omnivore	RFA
312	*Sufflamen fraenatum*	LC	Omnivore	RFA;OD
	Diodontidae			
	**Diodon**			
313	*Diodon hystrix*	LC	Carnivore	RFA
314	*Diodon liturosus*	NE	Carnivore	RFA
	Monacanthidae			
	**Aluterus**			
315	*Aluterus scriptus*	LC	Herbivore	RFA

No.	Class, Order, Family, Genus, Species	IUCN	Feeding habits	Habitat
	**Cantherhines**			
316	*Cantherhines dumerilii*	LC	Omnivore	RFA
317	*Cantherhines pardalis*	LC	Omnivore	RFA
	Ostraciidae			
	**Ostracion**			
318	*Ostracion cubicus*	NE	Omnivore	RFA
	Tetraodontidae			
	**Arothron**			
319	*Arothron hispidus*	LC	Omnivore	BRA;RFA
320	*Arothron mappa*	LC	Omnivore	RFA
321	*Arothron nigropunctatus*	LC	Omnivore	RFA
322	*Arothron stellatus*	LC	Omnivore	BRA;RFA
	**Chondrichthyes**			
	**Carcharhiniformes**			
	Carcharhinidae			
	**Carcharhinus**			
323	*Carcharhinus amblyrhynchoides*	NT	Carnivore	PE
324	*Carcharhinus falciformis*	VU	Carnivore	RFA;OD
325	*Carcharhinus longimanus*	CR	Carnivore	PELO;OD
	**Galeocerdo**			
326	*Galeocerdo cuvier*	NT	Carnivore	BRA;BEP;OD
	**Glyphis**			
327	*Glyphis gangeticus*	CR	Carnivore	BRA;DEM;AMP
	Triakidae			
	**Mustelus**			
328	*Mustelus griseus*	EN	Carnivore	DEM
	**Hexanchiformes**			
	Hexanchidae			
	**Hexanchus**			
329	*Hexanchus griseus*	NT	Carnivore	BAD;OD
	**Myliobatiformes**			
	Dasyatidae			
	**Dasyatis**			
330	*Dasyatis sinensis*	EN	Carnivore	DEM
	**Taeniura**			
331	*Taeniura meyeni*	VU	Carnivore	RFA
	**Urogymnus**			
332	*Urogymnus asperrimus*	VU	Carnivore	BRA;RFA
	Gymnuridae			
	**Gymnura**			
333	*Gymnura japonica*	VU	Carnivore	DEM
	**Rajiformes**			
	Rhynchobatidae			

No.	Class, Order, Family, Genus, Species	IUCN	Feeding habits	Habitat
	**Rhynchobatus**			
334	*Rhynchobatus djiddensis*	CR	Carnivore	BRA;RFA
	**Squaliformes**			
	Squalidae			
	**Squalus**			
335	*Squalus brevirostris*	EN	Carnivore	DEM
336	*Squalus japonicus*	EN	Carnivore	BAD

Note: The Habitats are indicated as: Brackish (BRA); reef-associated (RFA); oceanodromous (OD); amphidromous (AMP); anadromous (ANA); pelagic-neritic (PE); bathypelagic (BAP); benthopelagic (BEP); pelagic-oceanic (PELO); pelagic (PEL); demersal (DEM); bathydemersal(BAD); Marine (MAR); freshwater (FRE); potamodromous (POT). The IUCN status are indicated as: Critically Endangered (CR); Endangered (EN); Vulnerable (VU); Near Threatened (NT); Least Concern (LC); Data Deficient (DD); Not Evaluated (NE).
